# Assessment of Primary Malignancy Risk after Initiation of Biologic Therapy in Patients with Psoriasis

**DOI:** 10.1016/j.xjidi.2025.100397

**Published:** 2025-07-04

**Authors:** Chunghwan Ro, Ana Ormaza Vera, Waleed Adawi, Alexander Yap, Clinton W. Enos

**Affiliations:** 1 Department of Dermatology, Eastern Virginia Medical School, Macon & Joan Brock Virginia Health Sciences at Old Dominion University, Norfolk, Virginia, USA; 2 TriNetX, LLC, Cambridge, Massachusetts, USA

**Keywords:** Biologics, Epidemiology, Malignant melanoma, Nonmelanoma skin cancer, Oncology

## Abstract

Limited evidence exists regarding the long-term oncologic safety of biologic therapies, particularly IL-17 inhibitors and IL-23 inhibitors, in the management of psoriasis. This propensity score–matched retrospective cohort study assessed the risk of developing a primary malignancy within 10 years after exposure to a Food and Drug Administration–approved biologic among patients with psoriasis compared with that among biologic-naïve controls. After propensity score matching, 32,230 biologic-exposed patients were further grouped into cohorts of TNF inhibitors (n = 16,011), IL-23 inhibitors (n = 5604), IL-12/23 inhibitors (n =3856), and IL-17 inhibitors (n = 5467). During a 10-year period, TNF inhibitor–treated patients had a reduced risk of developing any primary malignancy compared with biologic-naïve patients with psoriasis (hazard ratio [HR] = 0.80; 95% confidence interval [CI] = 0.73–0.87). Similarly, a lower incidence of any malignancy was observed among patients on IL-23 inhibitors (HR = 0.83; 95% CI = 0.68–1.02), IL-12/23 inhibitors (HR = 0.85; 95% CI = 0.71–1.03), or IL-17 inhibitors (HR = 0.87; 95% CI = 0.73–1.04); however, the differences did not reach statistical significance. TNF inhibitor users were less likely to develop nonmelanoma skin cancer (HR = 0.82; 95% CI = 0.71–0.96) than controls, and the risk of nonmelanoma skin cancer did not significantly differ among users of IL-23 inhibitors (HR = 1.09; 95% CI = 0.77–1.55), IL-12/23 inhibitors (HR = 1.22; 95% CI = 0.90–1.64), or IL-17 inhibitors (HR = 1.03; 95% CI = 0.77–1.38). Exposure to any biologic class did not associate with the risk of developing melanoma or lymphoid/hematopoietic malignancies. Overall, these results provide evidence for the long-term oncologic safety of biologic therapies in the management of psoriasis.

## Introduction

Psoriasis has been associated with an elevated risk of certain malignancies relative to the general population (Trafford et al, 2019; Vaengebjerg et al, 2020), a link often attributed to chronic systemic inflammation, immune dysregulation, and lifestyle factors such as smoking and alcohol use (Boehncke and Schön, 2015). Although biologic therapies have revolutionized psoriasis management by significantly improving disease outcomes through targeted immunomodulation (Armstrong and Read, 2020), concerns remain regarding their long-term oncologic safety owing to their immunomodulatory effects (Briukhovetska et al, 2021). As a result, patients may experience apprehension initiating or continuing biologics owing to concerns about the potential long-term risk of malignancy (Lebwohl et al, 2016). Previous studies examining this association have yielded mixed results (Vaengebjerg et al, 2020), and there is limited evidence regarding the long-term oncologic safety profiles of newer biologic agents such as IL-17 inhibitor (IL-17i) and IL-23 inhibitor (IL-23i).

In this study, we conducted a propensity score–matched retrospective cohort analysis comparing patients with psoriasis with biologic exposure with biologic-naïve patients. Cohorts included patients with up to 10 years of follow-up, allowing for the assessment of both early- and late-onset malignancies. By encompassing a diverse group of biologic agents and extending the observation period, we aimed to provide a more comprehensive evaluation of the incidence and risk of primary malignancy associated with biologic treatments for psoriasis.

## Results

Before propensity score matching, 49,609 patients with psoriasis received biologic therapy, whereas 206,057 did not (Table 1). In the crude analysis, there was an observed decreased risk for primary malignancy risk in each biologic-class cohort (TNF-α inhibitor [TNFi], IL-17 inhibitor [IL-17i], IL-12/23 inhibitor [IL-12/23i], or IL-23 inhibitors [IL-23i]) compared with that in biologic-naïve controls (risk for all malignancy: TNFi hazard ratio [HR] = 0.69, 95% confidence interval [CI] = 0.65–0.73; IL-23i HR = 0.81, 95% CI = 0.71–0.93; IL-12/23i HR = 0.73, 95% CI = 0.64–0.84; IL-17i HR = 0.74, 95% CI = 0.66–0.83) (Table 2). After propensity score matching, a total of 32,230 biologic-treated patients with psoriasis were included in the analysis (Table 1). These were further categorized into 16,011 patients receiving only TNFi; 5604 initiating IL-23i exclusively; 3856 initiating IL-12/23i exclusively; and 5467 initiating IL-17i exclusively. Each treatment group was 1:1 propensity score matched to a biologic-naïve control cohort (Table 3, Table 4, Table 5, Table 6). The maximum duration of treatment was 9.96 years for TNFi, 9.26 years for IL-17i, 9.98 years for IL-12/23i, and 6.4 years for IL-23i (Table 7).

**Table 1:** Baseline Characteristics of Patients with Psoriasis with and without Biologic Therapies

Characteristic	Crude		Std diff.	Adjusted		Std diff.
	Psoriasis, Biologic (n = 49,609)	Psoriasis, No Biologic (n = 206,057)		Psoriasis, Biologic (n = 30,938)	Psoriasis, No Biologic (n = 30,938)	
Age at index, y, mean (SD)	48.1 (15)	51 (16.6)	0.19	47.7 (15.4)	48 (16)	0.02
Gender, n (%)						
Male	21,394 (43.1)	84,612 (41.1)	0.04	13,675 (44.2)	14,163 (45.8)	0.03
Female	26,023 (52.5)	108,403 (52.6)	0.00	15,946 (51.5)	15,720 (50.8)	0.01
Unknown gender	2192 (4.4)	13,042 (6.3)	0.08	1317 (4.3)	1055 (3.4)	0.04
Race, n (%)						
American Indian or Alaska Native	247 (0.5)	687 (0.3)	0.03	153 (0.5)	130 (0.4)	0.01
Asian	1830 (3.7)	10,354 (5.0)	0.07	1203 (3.9)	1150 (3.7)	0.01
Black or African American	2418 (4.9)	10,376 (5.1)	0.01	1750 (5.6)	1625 (5.3)	0.02
Native Hawaiian or Other Pacific Islander	131 (0.3)	803 (0.4)	0.02	85 (0.3)	80 (0.3)	0.00
Other race	1750 (3.5)	6303 (3.1)	0.03	1154 (3.7)	1086 (3.5)	0.01
Unknown race	6435 (12.9)	33,234 (16.1)	0.09	4138 (13.4)	3770 (12.2)	0.04
White	36,798 (74.2)	144,300 (70.0)	0.09	22,455 (72.6)	23,097 (74.6)	0.05
Ethnicity, n (%)						
Hispanic or Latino	3054 (6.2)	12,367 (6.0)	0.01	2007 (6.5)	1928 (6.2)	0.01
Not Hispanic or Latino	34,701 (69.9)	138,770 (67.3)	0.06	21,369 (69.1)	21,497 (69.5)	0.01
Unknown ethnicity	11,854 (23.9)	54,920 (26.7)	0.06	7561 (24.4)	7512 (24.3)	0.00
Diagnosis, n (%)						
Arthropathic psoriasis (L40.5)	20,488 (41.3)	5801 (2.8)	1.05	4771 (15.4)	5203 (16.8)	0.04
Essential (primary) hypertension (I10)	14,434 (29.1)	56,503 (27.4)	0.04	8587 (27.8)	8505 (27.5)	0.01
Overweight and obesity (E66)	10,292 (20.7)	30,613 (14.9)	0.15	5862 (18.9)	5913 (19.1)	0.00
Nicotine dependence (F17.2)	5857 (11.8)	19,140 (9.3)	0.08	3583 (11.6)	3453 (11.2)	0.01
Noninfective enteritis and colitis (K50–52)	4339 (8.7)	12,741 (6.2)	0.10	2628 (8.5)	2655 (8.6)	0.00
Alcohol related disorders (F10)	1899 (3.8)	5926 (2.9)	0.05	1197 (3.9)	1115 (3.6)	0.01
Unspecified viral hepatitis C (B19.2)	469 (0.9)	1581 (0.8)	0.02	293 (0.9)	290 (0.9)	0.00
HIV disease (B20)	143 (0.3)	880 (0.4)	0.02	92 (0.3)	89 (0.3)	0.00
Unspecified viral hepatitis B (B19.1)	146 (0.3)	449 (0.2)	0.02	85 (0.3)	71 (0.2)	0.01
Tuberculosis (A15–19)	152 (0.3)	289 (0.1)	0.04	81 (0.3)	58 (0.2)	0.02
Exposure to tanning bed (W89.1)	15 (0.0)	16 (0.0)	0.02	12 (0.0)	10 (0.0)	0.00
Medication, n (%)						
Methotrexate	11,206 (22.6)	6820 (3.3)	0.60	4024 (13.0)	4047 (13.1)	0.00
Apremilast	4920 (9.9)	1201 (0.6)	0.43	1144 (3.7)	1141 (3.7)	0.00
Cyclosporine	1315 (2.6)	1476 (0.7)	0.15	689 (2.2)	667 (2.2)	0.00
Azathioprine	463 (0.9)	888 (0.4)	0.06	246 (0.8)	253 (0.8)	0.00
Mycophenolate mofetil	230 (0.5)	838 (0.4)	0.01	161 (0.5)	145 (0.5)	0.01

Abbreviation: Std diff., Standardized mean difference.

**Table 2:** Crude Incidence and Risk of Primary Malignancy after Initiation of a Biologic in Patients with Psoriasis at 10 years

Drug Class	Outcomes	Incidences		Hazard Ratio (95% CI)	Risk Difference %	*P*-Value
		Biologic	Nonbiologic			
TNFi		n = 21,619	n = 206,057			
	All malignancy^1^	1091	17,094	0.69 (0.65–0.73)	−3.64%	<.001
	NMSC^2^	392	6128	0.74 (0.67–0.82)	−1.16%	<.001
	MM^3^	53	1092	0.56 (0.43–0.73)	−0.29%	<.001
	Lymphoid and hematopoietic^4^	98	1869	0.60 (0.50–0.74)	−0.45%	<.001
IL-23i		n = 6137	n = 206,057			
	All malignancy^1^	193	17,094	0.81 (0.71–0.93)	−5.54%	.002
	NMSC^2^	63	6128	0.82 (0.65–1.03)	−1.94%	.09
	MM^3^	11	1092	0.71 (0.39–1.29)	−0.35%	.26
	Lymphoid and hematopoietic^4^	22	1869	1.12 (0.60–2.09)	−0.55%	.49
IL-12/23i		n = 3890	n = 206,057			
	All malignancy^1^	207	17,094	0.73 (0.64–0.84)	−3.37%	<.001
	NMSC^2^	91	6128	0.96 (0.78–1.17)	−0.64%	.66
	MM^3^	16	1092	0.93 (0.57–1.53)	−0.12%	.78
	Lymphoid and hematopoietic^4^	19	1869	0.64 (0.41–1.00)	−0.42%	.05
IL-17i		n = 6536	n = 206,057			
	All malignancy^1^	261	17 094	0.74 (0.66–0.83)	−4.69%	<.001
	NMSC^2^	94	6128	0.81 (0.66–0.98)	−1.53%	.03
	MM^3^	18	1092	0.90 (0.58–1.40)	−0.25%	.63
	Lymphoid and hematopoietic^4^	46	1869	1.26 (0.95–1.67)	−0.20%	.10

Abbreviations: CI, confidence interval; ICD-10, International Classification of Diseases, Tenth Revision; IL-17i, IL-17 inhibitor; IL-12/23i, IL-12/23 inhibitor; IL-23i, IL-23 inhibitor; MM, malignant melanoma; NMSC, nonmelanoma skin cancer; TNFi, TNF inhibitor.

1 ICD-10: C00-D09.

2 NMSC ICD-10: C44, C4A, and D04.

3 MM ICD-10: C43 and D03.

4 ICD-10: C81–C96.

**Table 3:** Baseline Characteristics of Patients with Psoriasis on TNFi and without Biologic Therapies

Characteristic	Crude		Std diff.	Adjusted		Std diff.
	TNFi (n = 21,619)	Nonbiologic (n = 206,057)		TNFi (n = 16,011)	Nonbiologic (n = 16,011)	
Age at index, y, mean (SD)	48.3 (15.3)	51 (16.6)	0.15	48.7 (15.5)	49.1 (16.2)	0.03
Gender, n (%)						
Male	9426 (43.6)	84,612 (41.1)	0.05	7029 (43.9)	7403 (46.2)	0.05
Female	11,349 (52.5)	108,403 (52.6)	0.01	8350 (52.1)	8151 (50.9)	0.02
Unknown gender	844 (3.9)	13,042 (6.3)	0.09	632 (4.0)	456 (2.9)	0.06
Race, n (%)						
American Indian or Alaska Native	130 (0.6)	687 (0.3)	0.04	90 (0.6)	88 (0.6)	0.00
Asian	631 (2.9)	10,354 (5.0)	0.12	540 (3.4)	573 (3.6)	0.01
Black or African American	1116 (5.2)	10,376 (5.1)	0.01	898 (5.6)	877 (5.5)	0.01
Native Hawaiian or Other Pacific Islander	55 (0.3)	803 (0.4)	0.10	48 (0.3)	40 (0.3)	0.01
Other race	847 (3.9)	6303 (3.1)	0.02	634 (4.0)	596 (3.7)	0.01
Unknown race	2503 (11.6)	33,234 (16.1)	0.10	1928 (12.0)	1692 (10.6)	0.05
White	16,337 (75.6)	144,300 (70.0)	0.14	11,872 (74.2)	12,144 (75.9)	0.04
Ethnicity, n (%)						
Hispanic or Latino	1621 (7.5)	12,367 (6.0)	0.03	1207 (7.5)	1206 (7.5)	0.00
Not Hispanic or Latino	15,078 (69.7)	138,770 (67.3)	0.04	11,072 (69.1)	11,297 (70.6)	0.03
Unknown ethnicity	4920 (22.8)	54,920 (26.7)	0.06	3732 (23.3)	3508 (21.9)	0.03
Diagnosis, n (%)						
Arthropathic psoriasis (L40.5)	10,878 (50.3)	5801 (2.8)	1.25	5256 (32.8)	5298 (33.1)	0.01
Essential (primary) hypertension (I10)	6188 (28.6)	56,503 (27.4)	0.02	4504 (28.1)	4590 (28.7)	0.01
Overweight and obesity (E66)	4314 (20.0)	30,613 (14.9)	0.12	2904 (18.1)	2882 (18.0)	0.00
Nicotine dependence (F17.2)	2425 (11.2)	19 140 (9.3)	0.06	1676 (10.5)	1752 (10.9)	0.02
Noninfective enteritis and colitis (K50–52)	2021 (9.3)	12 741 (6.2)	0.11	1401 (8.8)	1511 (9.4)	0.02
Alcohol related disorders (F10)	846 (3.9)	5926 (2.9)	0.05	612 (3.8)	612 (3.8)	0.00
Unspecified viral hepatitis C (B19.2)	216 (1.0)	1581 (0.8)	0.03	157 (1.0)	155 (1.0)	0.00
HIV disease (B20)	57 (0.3)	880 (0.4)	0.00	42 (0.3)	48 (0.3)	0.01
Unspecified viral hepatitis B (B19.1)	64 (0.3)	449 (0.2)	0.03	51 (0.3)	51 (0.3)	0.00
Tuberculosis (A15–19)	57 (0.3)	289 (0.1)	0.02	37 (0.2)	37 (0.2)	0.00
Exposure to tanning bed (W89.1)	10 (0.0)	16 (0.0)	0.02	10 (0.1)	10 (0.1)	0.00
Medication, n (%)						
Methotrexate	5526 (25.6)	6820 (3.3)	0.66	2416 (15.1)	3032 (18.9)	0.10
Apremilast	1503 (7.0)	1201 (0.6)	0.32	663 (4.1)	738 (4.6)	0.02
Cyclosporine	423 (2.0)	1476 (0.7)	0.09	291 (1.8)	328 (2.0)	0.02
Azathioprine	90 (0.4)	888 (0.4)	0.00	70 (0.4)	90 (0.6)	0.02
Mycophenolate mofetil	208 (1.0)	838 (0.4)	0.06	144 (0.9)	144 (0.9)	0.00

Abbreviations: Std diff., Standardized mean difference; TNFi, TNF inhibitor.

**Table 4:** Baseline Characteristics of Patients with Psoriasis on IL-23i and without Biologic Therapies

Characteristic	Crude		Std diff.	Adjusted		Std diff.
	IL-23i (n = 6137)	Nonbiologic (n = 206,057)		IL-23i (n = 5604)	Nonbiologic (n = 5604)	
Age at index, y, mean (SD)	49.0 (15.3)	51 (16.6)	0.11	48.8 (15.4)	49.2 (15.8)	0.03
Gender, n (%)						
Male	2828 (46.1)	84,612 (41.1)	0.10	2592 (46.3)	2629 (46.9)	0.01
Female	3131 (51.0)	108,403 (52.6)	0.04	2853 (50.9)	2854 (50.9)	0.00
Unknown G=gender	178 (2.9)	13,042 (6.3)	0.14	159 (2.8)	121 (2.2)	0.04
Race, n (%)						
American Indian or Alaska Native	22 (0.4)	687 (0.3)	0.00	19 (0.3)	10 (0.2)	0.03
Asian	292 (4.8)	10,354 (5.0)	0.03	264 (4.7)	237 (4.2)	0.02
Black or African American	347 (5.7)	10,376 (5.1)	0.02	316 (5.6)	345 (6.2)	0.02
Native Hawaiian or Other Pacific Islander	21 (0.3)	803 (0.4)	0.09	18 (0.3)	13 (0.2)	0.02
Other race	274 (4.5)	6303 (3.1)	0.05	261 (4.7)	227 (4.1)	0.03
Unknown race	722 (11.8)	33,234 (16.1)	0.09	665 (11.9)	592 (10.6)	0.04
White	4459 (72.7)	144,300 (70.0)	0.06	4061 (72.5)	4180 (74.6)	0.05
Ethnicity, n (%)						
Hispanic or Latino	342 (5.6)	12,367 (6.0)	0.05	313 (5.6)	252 (4.5)	0.05
Not Hispanic or Latino	4357 (71.0)	138,770 (67.3)	0.06	3952 (70.5)	4055 (72.4)	0.04
Unknown ethnicity	1438 (23.4)	54 920 (26.7)	0.04	1339 (23.9)	1297 (23.1)	0.02
Diagnosis, n (%)						
Arthropathic psoriasis (L40.5)	1145 (18.7)	5801 (2.8)	0.50	769 (13.7)	679 (12.1)	0.05
Essential (primary) hypertension (I10)	1985 (32.3)	56,503 (27.4)	0.10	1747 (31.2)	1751 (31.2)	0.00
Overweight and obesity (E66)	1594 (26.0)	30,613 (14.9)	0.25	1399 (25.0)	1351 (24.1)	0.02
Nicotine dependence (F17.2)	881 (14.4)	19,140 (9.3)	0.15	760 (13.6)	727 (13.0)	0.02
Noninfective enteritis and colitis (K50–52)	533 (8.7)	12,741 (6.2)	0.08	475 (8.5)	443 (7.9)	0.02
Alcohol related disorders (F10)	297 (4.8)	5926 (2.9)	0.09	260 (4.6)	236 (4.2)	0.02
Unspecified viral hepatitis C (B19.2)	49 (0.8)	1581 (0.8)	0.00	45 (0.8)	36 (0.6)	0.02
HIV disease (B20)	21 (0.3)	880 (0.4)	0.02	17 (0.3)	16 (0.3)	0.00
Unspecified viral hepatitis B (B19.1)	23 (0.4)	449 (0.2)	0.01	20 (0.4)	16 (0.3)	0.01
Tuberculosis (A15–19)	22 (0.4)	289 (0.1)	0.04	14 (0.3)	27 (0.5)	0.04
Exposure to tanning bed (W89.1)	10 (0.2)	16 (0.0)	0.05	10 (0.2)	10 (0.2)	0.00
Medication, n (%)						
Methotrexate	686 (11.2)	6820 (3.3)	0.59	533 (9.5)	554 (9.9)	0.01
Apremilast	1022 (16.7)	1201 (0.6)	0.30	653 (11.7)	677 (12.1)	0.01
Cyclosporine	197 (3.2)	1476 (0.7)	0.17	161 (2.9)	155 (2.8)	0.01
Azathioprine	29 (0.5)	888 (0.4)	0.00	26 (0.5)	22 (0.4)	0.01
Mycophenolate mofetil	38 (0.6)	838 (0.4)	0.03	32 (0.6)	22 (0.4)	0.03

Abbreviations: IL-23i, IL-23 inhibitor; Std diff., Standardized mean difference.

**Table 5:** Baseline Characteristics of Patients with Psoriasis on IL-12/23i and without Biologic Therapies

Characteristics	Crude		Std diff.	Adjusted		Std diff.
	IL-12/23i (n = 3890)	Nonbiologic (n = 206,057)		IL-12/23i (n = 3856)	Nonbiologic (n = 3856)	
Age at index, y, mean (SD)	46.8 (16.5)	51 (16.6)	0.24	46.9 (16.5)	47.1 (16.6)	0.02
Gender, n (%)						
Male	1767 (45.4)	84,612 (41.1)	0.09	1751 (45.4)	1782 (46.2)	0.02
Female	2005 (51.5)	108,403 (52.6)	0.03	1986 (51.5)	1981 (51.4)	0.00
Unknown gender	119 (3.1)	13,042 (6.3)	0.14	119 (3.1)	93 (2.4)	0.04
Race, n (%)						
American Indian or Alaska Native	11 (0.3)	687 (0.3)	0.02	10 (0.3)	12 (0.3)	0.01
Asian	198 (5.1)	10,354 (5.0)	0.02	197 (5.1)	170 (4.4)	0.03
Black or African American	233 (6.0)	10,376 (5.1)	0.03	232 (6.0)	232 (6.0)	0.00
Native Hawaiian or Other Pacific Islander	11 (0.3)	803 (0.4)	0.10	10 (0.3)	10 (0.3)	0.00
Other race	173 (4.4)	6303 (3.1)	0.06	170 (4.4)	171 (4.4)	0.00
Unknown race	536 (13.8)	33,234 (16.1)	0.04	531 (13.8)	488 (12.7)	0.03
White	2735 (70.3)	144,300 (70.0)	0.02	2706 (70.1)	2773 (71.9)	0.04
Ethnicity, n (%)						
Hispanic or Latino	252 (6.5)	138,770 (67.3)	0.00	249 (6.5)	213 (5.5)	0.04
Not Hispanic or Latino	2621 (67.4)	12,367 (6.0)	0.01	2597 (67.3)	2615 (67.8)	0.01
Unknown ethnicity	1017 (26.1)	54,920 (26.7)	0.01	1011 (26.2)	1028 (26.7)	0.01
Diagnosis, n (%)						
Arthropathic psoriasis (L40.5)	848 (21.8)	5801 (2.8)	0.58	818 (21.2)	805 (20.9)	0.01
Essential (primary) hypertension (I10)	1068 (27.5)	56,503 (27.4)	0.01	1053 (27.3)	1049 (27.2)	0.00
Overweight and obesity (E66)	763 (19.6)	30,613 (14.9)	0.10	754 (19.5)	760 (19.7)	0.00
Nicotine dependence (F17.2)	569 (14.6)	19,140 (9.3)	0.27	555 (14.4)	606 (15.7)	0.04
Noninfective enteritis and colitis (K50–52)	426 (10.9)	12,741 (6.2)	0.05	422 (11.0)	415 (10.8)	0.01
Alcohol related disorders (F10)	149 (3.8)	5926 (2.9)	0.04	146 (3.8)	138 (3.6)	0.01
Unspecified viral hepatitis C (B19.2)	24 (0.6)	1581 (0.8)	0.02	24 (0.6)	24 (0.6)	0.00
HIV disease (B20)	14 (0.4)	880 (0.4)	0.02	13 (0.3)	20 (0.5)	0.02
Unspecified viral hepatitis B (B19.1)	18 (0.5)	449 (0.2)	0.00	18 (0.5)	25 (0.6)	0.03
Tuberculosis (A15–19)	11 (0.3)	289 (0.1)	0.03	12 (0.3)	10 (0.3)	0.01
Exposure to tanning bed (W89.1)	11 (0.3)	16 (0.0)	0.07	10 (0.3)	10 (0.3)	0.00
Medication, n (%)						
Methotrexate	579 (14.9)	6820 (3.3)	0.41	558 (14.5)	628 (16.3)	0.05
Apremilast	368 (9.5)	1201 (0.6)	0.41	340 (8.8)	361 (9.4)	0.02
Cyclosporine	132 (3.4)	1476 (0.7)	0.18	128 (3.3)	145 (3.7)	0.02
Azathioprine	57 (1.5)	888 (0.4)	0.11	55 (1.4)	57 (1.5)	0.00
Mycophenolate mofetil	30 (0.8)	838 (0.4)	0.05	30 (0.8)	33 (0.9)	0.01

Abbreviations: IL-12/23i, IL-12/23 inhibitor; Std diff., Standardized mean difference.

**Table 6:** Baseline Characteristics of Patients with Psoriasis on IL-17i and without Biologic Therapies

Characteristics	Crude		Std diff.	Adjusted		Std diff.
	IL-17i (n = 6536)	Nonbiologic (n = 206,057)		IL-17i (n = 5467)	Nonbiologic (n = 5467)	
Age at index, y, mean (SD)	49.9 (14.3)	51 (16.6)	0.21	49.9 (14.4)	51.1 (15.4)	0.08
Gender, n (%)						
Male	2847 (43.6%)	84,612 (41.1%)	0.05	2416 (44.2%)	2493 (45.6%)	0.03
Female	3384 (51.8%)	108,403 (52.6%)	0.03	2811 (51.4%)	2791 (51.1%)	0.01
Unknown gender	305 (4.6%)	13,042 (6.3%)	0.04	240 (4.4%)	183 (3.3%)	0.05
Race, n (%)						
American Indian or Alaska Native	27 (0.4)	687 (0.3)	0.01	23 (0.4)	14 (0.3)	0.03
Asian	294 (4.5)	10,354 (5.0)	0.03	257 (4.7)	248 (4.5)	0.01
Black or African American	333 (5.1)	10,376 (5.1)	0.00	288 (5.3)	264 (4.8)	0.02
Native Hawaiian or Other Pacific Islander	19 (0.3)	803 (0.4)	0.09	15 (0.3)	11 (0.2)	0.02
Other race	276 (4.2)	6303 (3.1)	0.05	220 (4.0)	194 (3.5)	0.02
Unknown Race	988 (15.1)	33,234 (16.1)	0.03	816 (14.9)	723 (13.2)	0.05
White	4599 (70.4)	144,300 (70.0)	0.01	3848 (70.4)	4013 (73.4)	0.07
Ethnicity, n (%)						
Hispanic or Latino	417 (6.4)	138,770 (67.3)	0.01	351 (6.4)	305 (5.6)	0.04
Not Hispanic or Latino	4269 (65.3)	12,367 (6.0)	0.08	3606 (66.0)	3713 (67.9)	0.04
Unknown ethnicity	1850 (28.3)	54,920 (26.7)	0.09	1510 (27.6)	1449 (26.5)	0.03
Diagnosis, n (%)						
Arthropathic psoriasis (L40.5)	2735 (41.8%)	5801 (2.8%)	1.04	1678 (30.7%)	1641 (30.0%)	0.01
Essential (primary) hypertension (I10)	2164 (33.1%)	56,503 (27.4%)	0.11	1764 (32.3%)	1837 (33.6%)	0.03
Overweight and obesity (E66)	1517 (23.2%)	30,613 (14.9%)	0.01	1200 (22.0%)	1166 (21.3%)	0.02
Nicotine dependence (F17.2)	827 (12.7%)	19,140 (9.3%)	0.08	651 (11.9%)	599 (11.0%)	0.03
Noninfective enteritis and colitis (K50–52)	368 (5.6%)	12,741 (6.2%)	0.10	298 (5.5%)	284 (5.2%)	0.01
Alcohol related disorders (F10)	280 (4.3%)	5926 (2.9%)	0.05	226 (4.1%)	206 (3.8%)	0.02
Unspecified viral hepatitis C (B19.2)	51 (0.8%)	1581 (0.8%)	0.02	45 (0.8%)	42 (0.8%)	0.01
HIV disease (B20)	21 (0.3%)	880 (0.4%)	0.02	15 (0.3%)	10 (0.2%)	0.02
Unspecified viral hepatitis B (B19.1)	20 (0.3%)	449 (0.2%)	0.01	16 (0.3%)	22 (0.4%)	0.02
Tuberculosis (A15–19)	17 (0.3%)	289 (0.1%)	0.03	13 (0.2%)	17 (0.3%)	0.01
Exposure to tanning bed (W89.1)	10 (0.2%)	16 (0.0%)	0.05	10 (0.2%)	10 (0.2%)	0.00
Medication, n (%)						
Methotrexate	1086 (16.6%)	6820 (3.3%)	0.45	707 (12.9%)	758 (13.9%)	0.03
Apremilast	1000 (15.3%)	1201 (0.6%)	0.56	517 (9.5%)	616 (11.3%)	0.06
Cyclosporine	206 (3.2%)	1476 (0.7%)	0.17	144 (2.6%)	161 (2.9%)	0.02
Azathioprine	38 (0.6%)	888 (0.4%)	0.02	26 (0.5%)	27 (0.5%)	0.00
Mycophenolate mofetil	48 (0.7%)	838 (0.4%)	0.04	36 (0.7%)	33 (0.6%)	0.01

Abbreviations: IL-17i, IL-17 inhibitor; Std diff., Standardized mean difference.

**Table 7:** Time on Biologic Treatment for Patients with Psoriasis^1^

Drug	Time on Treatment, y		
	Mean (SD)	Median	Maximum
TNFi	3.44 (2.58)	2.95	9.96
IL-23i	1.60 (1.38)	1.39	6.4
IL-12/23i	3.42 (2.50)	2.98	9.98
IL-17i	2.45 (1.50)	1.46	9.26

Abbreviations: IL-17i, IL-17 inhibitor; IL-12/23i, IL-12/23 inhibitor; IL-23i, IL-23 inhibitor; TNFi, TNF inhibitor.

1 At baseline.

Within a 10-year observation window, TNFi-treated patients with psoriasis exhibited a reduced risk of any primary malignancy compared with the biologic-naïve controls (832 vs 1173, HR = 0.80, 95% CI= 0.73–0.87). Similar reduced risks were observed for NMSC (292 vs 392, HR = 0.82, 95% CI = 0.71–0.96). Although reduced incidences of malignant melanoma (41 vs 59, HR = 0.72, 95% CI = 0.49–1.06) and lymphoid or hematopoietic cancers (84 vs 126, HR = 0.78, 95% CI = 0.60–1.02) were observed, these did not reach statistical significance (Table 8).

**Table 8:** Incidence and Risk of Primary Malignancy after Initiation of a Biologic in Patients with Psoriasis 10 Years after Propensity Score Matching

Drug Class	Outcomes	Incidences		Hazard Ratio (95% CI)	Risk Difference %	*P*-Value
		Biologic	Nonbiologic			
TNFi		n = 16,011	n = 16 011			
	All malignancy^1^	832	1173	0.80 (0.73–0.87)	−2.31%	<.001
	NMSC^2^	292	392	0.82 (0.71–0.96)	−0.68%	.01
	MM^3^	41	59	0.72 (0.49–1.06)	−0.12%	.26
	Lymphoid and hematopoietic^4^	84	126	0.78 (0.60–1.02)	−0.28%	.06
IL-23i		n = 5604	n = 5604			
	All malignancy^1^	174	209	0.83 (0.68–1.02)	−0.63%	.07
	NMSC^2^	60	56	1.09 (0.77–1.55)	0.07%	.62
	MM^3^	10	11	0.90 (0.38–2.12)	−0.02%	.81
	Lymphoid and hematopoietic^4^	21	19	1.12 (0.60–2.09)	0.04%	.72
IL-12/23i		n = 3856	n = 3856			
	All malignancy^1^	199	257	0.85 (0.71–1.03)	−1.55%	.09
	NMSC^2^	90	84	1.22 (0.90–1.64)	0.16%	.21
	MM^3^	13	18	0.80 (0.39–1.64)	−0.13%	.54
	Lymphoid and hematopoietic^4^	18	29	0.70 (0.39–1.26)	−0.30%	.23
IL-17i		n = 5467	n = 5467			
	All malignancy^1^	189	235	0.87 (0.73–1.04)	−1.04%	.12
	NMSC^2^	67	57	1.03 (0.77–1.38)	0.23%	.85
	MM^3^	17	20	0.91 (0.48–1.75)	−0.07%	.78
	Lymphoid and hematopoietic^4^	39	26	1.56 (0.95–2.58)	0.29%	.08

Abbreviations: CI, confidence interval; ICD-10, International Classification of Diseases, Tenth Revision; IL-17i, IL-17 inhibitor; IL-12/23i, IL-12/23 inhibitor; IL-23i, IL-23 inhibitor; MM, malignant melanoma; NMSC, nonmelanoma skin cancer; TNFi, TNF inhibitor.

1 ICD-10: C00-D09.

2 NMSC ICD-10: C44, C4A, and D04.

3 MM ICD-10: C43 and D03.

4 ICD-10: C81–C96.

Decreased incidence of developing any primary malignancy was observed for those treated with IL-23i (174 vs 209, HR = 0.83, 95% CI = 0.68–1.02), IL-12/23i (199 vs 257, HR = 0.85, 95% CI = 0.71–1.03), or IL-17i (189 vs 235, HR = 0.87, 95% CI = 0.73–1.04) compared with that for the biologic-naïve controls; however, CIs included the null. There was no significant difference in the risk of developing NMSC, malignant melanoma, and lymphoid or hematopoietic cancers compared with the controls over the same period for these cohorts (Table 8).

## Discussion

In this retrospective cohort study, biologic therapies were not associated with an increased risk of primary malignancy in patients with psoriasis over a 10-year follow-up period. Notably, TNFi-treated patients with psoriasis showed a reduced risk of NMSC, diverging from prior findings (Asgari et al, 2017; Peleva et al, 2018), offering reassurance regarding their oncologic safety. The divergence between our findings and prior data may stem from differences in study populations, follow-up durations, and methodological approaches.

Recent evidence suggests that IL-17i and IL-23i do not confer an elevated malignancy risk compared with TNFi and with matched biologic-naïve patients (Kridin et al, 2024). Although this study has been instrumental in establishing confidence in the safety profiles of IL-17i and IL-23i, the shorter observation period may limit detection of potential late-emerging malignancies beyond 5 years. This analysis not only confirms prior findings (Kridin et al, 2024; Wu et al, 2023) but also reinforces the reassuring profile of these novel agents beyond 5 years through an extended follow-up period of up to 10 years.

Our data support the role of TNFi, IL-17i, IL-12/23i, and IL-23i in the long-term clinical management of psoriasis, offering greater reassurance to both clinicians and patients in decisions regarding sustained biologic therapy.

Limitations include differences in treatment availability and follow-up durations across biologic subclasses, which may have influenced the results. Potential inaccuracies in electronic medical records used to source data also may affect diagnostic precision and event reporting. In addition, matching on patient-level data alone may not fully address center-level confounding, and TriNetX’s open system might miss diagnoses or treatments occurring elsewhere. Consequently, data should be interpreted as purely observational without establishing a cause–effect relationship between biological therapies and the incidence of primary malignancies.

In conclusion, biologic therapies were not associated with an increased risk of primary malignancies in patients with psoriasis during a 10-year follow-up period; in fact, we often observe a decreased risk for malignancy. Notably, TNFi demonstrated a reduced risk of NMSC, challenging prior data and providing reassurance regarding its oncologic safety profile. Ultimately, the prescription of biologics should be approached thoughtfully and tailored to the individual needs of each patient.

## Materials and Methods

This observational retrospective cohort study utilized deidentified data from the federated electronic health record TriNetX from 2014 to 2024. TriNetX is a global research platform that provides real-time access to data from a range of healthcare organizations, including hospitals, outpatient clinics, academic medical centers, and community hospitals. This study utilized data from the TriNetX Research Network, which encompasses more than 133 million individuals receiving care at 95 healthcare organizations located in multiple countries but primarily focused on the United States. The data included demographic information, diagnoses (documented according to ICD-10, Clinical Modification), procedures (recorded using either the ICD-10, Procedure Coding System; Current Procedural Terminology; or Healthcare Common Procedure Coding System), medications, and laboratory test results.

Patients aged 18–89 years with 2 separate psoriasis diagnosis who initiated a TNFi (adalimumab, etanercept, infliximab, certolizumab pegol), IL-17i (secukinumab, ixekizumab, brodalumab), IL-12/23i (ustekinumab), or IL-23i (guselkumab, Risankizumab, and tildrakizumab) between September 2014 and September 2024 were included. Individuals with a known history of malignancy prior to their first psoriasis diagnosis were excluded from analysis. The control cohort included patients with psoriasis with similar eligibility criteria without biologic exposure. To reduce the confounding impact of multiple therapeutic regimens, exclusion criteria ensured that individuals in any given treatment group had neither prior nor future exposure to any other biologic class. For example, participants in the IL-17i group could not have ever received IL-23i, IL-12/23i, or TNFi.

**Figure 1:**
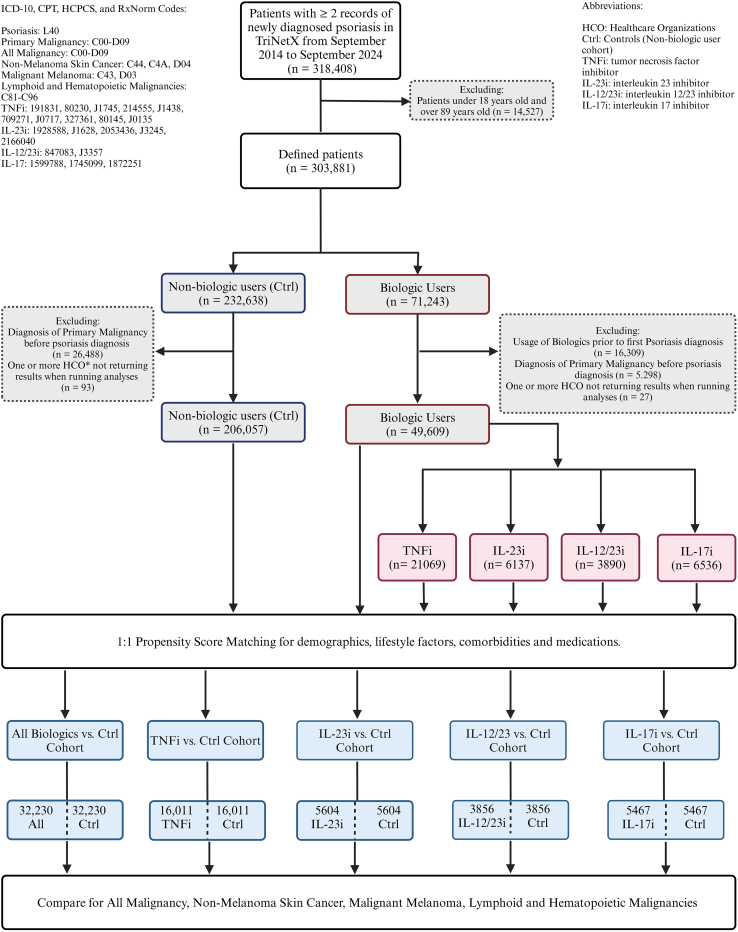
**Study design.** Shown is a flowchart illustrating the study design and cohort construction from the TriNetX database, with cohorts defined using ICD-10, CPT, HCPCS, and RxNorm codes. CPT, Current Procedural Terminology; Ctrl, control; HCO, healthcare organization; HCPCS, Healthcare Common Procedure Coding System; ICD, International Classification of Diseases; IL-17i, IL-17 inhibitor; IL-12/23i, IL-12/23 inhibitor; IL-23i, IL-23 inhibitor; TNFi, TNF inhibitor.

Of note, during the study period, International Classification of Diseases, Ninth Revision codes were utilized to identify patients and outcomes until the transition to ICD-10 coding in October 2015. TriNetX inherently addresses this coding transition by mapping International Classification of Diseases, Ninth Revision codes directly to their corresponding ICD-10 codes. Consequently, we included cases identified through International Classification of Diseases, Ninth Revision that seamlessly transitioned to ICD-10 within the analysis, ensuring the uniqueness of each patient case and eliminating any duplicate counting from patients diagnosed with both coding systems. The study design and relevant codes are depicted in Table 9 and Figure 1.

**Table 9:** ICD-10-CM Codes Utilized within TriNetX

ICD-10-CM Codes	Descriptor
L40	Psoriasis
C00-D09	Malignant neoplasms and in situ neoplasms
F10	Alcohol related disorders
F17.2	Nicotine dependence
W89.1	Exposure to tanning bed
E66	Overweight and obesity
L40.5	Arthropathic psoriasis
I10	Essential (primary) hypertension
A15–19	Tuberculosis
B20	HIV disease
B19.1	Unspecified viral hepatitis B
B19.2	Unspecified viral hepatitis C
K50–52	Noninfective enteritis and colitis
C44	Other and unspecified malignant neoplasms of the skin
C4A	Merkel cell carcinoma
D04	Carcinoma in situ of the skin
C43	Malignant melanoma of the skin
D03	Melanoma in situ
C81–C96	Malignant neoplasms of lymphoid, hematopoietic, and related tissues

Abbreviation: ICD-10-CM, International Classification of Diseases, Tenth Revision, Clinical Modification.

To account for potential confounding factors, we implemented 1:1 propensity score matching to create cohorts with comparable baseline characteristics. Matching characteristics included demographics (age, sex, race, and ethnicity), lifestyle factors (alcohol-related disorders, nicotine dependence, and exposure to tanning beds), comorbidities (overweight and obesity, arthropathic psoriasis, primary hypertension, tuberculosis, HIV disease, unspecific viral hepatitis B, unspecific viral hepatitis C, and noninfective enteritis and colitis), and medication use (methotrexate, apremilast, cyclosporine, azathioprine, and mycophenolate mofetil) (Table 1). Race and ethnicity classifications were derived from patient electronic medical records, which contain either self-reported data or classifications observed by healthcare providers. The race category labeled "Other Race" included patients who identified with multiple races or with a racial group not listed in the predefined database options (American Indian or Alaska Native, Asian, Black or African American, Native Hawaiian or Other Pacific Islander, unknown race, and White). The "Unknown Race" category was used when race information was not available within patient records.

Propensity score matching ensured an even distribution of confounding variables across the study groups. Propensity score matching was performed through a 1:1 matching with a nearest-neighbor greedy algorithm and a caliper set at 0.25 times the SD.

Cohorts were followed up to 10 years. HRs and 95% CIs were calculated to assess the risk of primary malignancy after treatment with biologics. Primary malignancies were defined according to ICD-10-CM codes as follows: all malignancies (C00-D09), NMSCs (C44, C4A, and D04), malignant melanoma (C43 and D03), and lymphoid and hematopoietic malignancies (C81–C96).

All study variables were reported as means with SDs for continuous variables and as frequencies with corresponding percentages for dichotomous variables. Comparisons involving continuous variables were conducted using Student’s *t*-test, whereas Pearson’s chi-square test was applied for dichotomous variables. Survival analyses were performed using the Kaplan–Meier method, and a log-rank test was used to determine differences in malignancy risk between the groups. HRs were calculated through Cox regression, and Schoenfeld residuals analysis was employed to assess the proportional hazards assumption. Statistical significance was defined as a 2-tailed *P* < .05. All statistical analyses, including propensity score matching, were conducted within the built-in TriNetX Compare Outcomes Analytics function (TriNetX, 2025).

## Ethics Statement

This study utilized deidentified patient data from TriNetX and is exempt from institutional review board approval.

## Data Availability Statement

The data underlying this article are available within the main text. All data were retrieved from TriNetX, LLC, a platform ensuring compliance with the Health Insurance Portability and Accountability Act Security Rule for the protection of healthcare data. Because all patient data were deidentified, this study was deemed exempt for institutional review board review.

## ORCIDs

Chunghwan Ro: http://orcid.org/0009-0000-3072-6732

Ana Ormaza Vera: http://orcid.org/0009-0004-4995-841X

Waleed Adawi: http://orcid.org/0000-0003-1242-7960

Alexander Yap: http://orcid.org/0000-0001-5695-9227

Clinton Enos: http://orcid.org/0000-0002-6783-1904

## Conflict of Interest

CE has previously served as a consultant on an advisory board for UCB and Amgen, is an investigator for Amgen and Castle Biosciences, and has previously received research funding from La Roche-Posay and the ASA/Arcutis Biotherapeutics. The remaining authors state no conflict of interest.
